# Establishment and field validation of a rapid on-site recombinase polymerase amplification–lateral flow assay for BRSV and BVDV

**DOI:** 10.3389/fvets.2026.1754704

**Published:** 2026-02-27

**Authors:** Zhiteng Zhao, Yanbing Guo, Shaoxiong Liu, Liangyu Hao, Xingzhong Sun, Yu Xiang, Nan Wang, Xiangyu Meng, Hongbo Sun, Shuai Yue, Pengtao Gong, Lili Cao

**Affiliations:** 1 State Key Laboratory for Diagnosis and Treatment of Severe Zoonotic Infectious Diseases, Key Laboratory for Zoonosis Research of the Ministry of Education, Institute of Zoonosis, and College of Veterinary Medicine, Jilin University, Changchun, China; 2 Jilin Academy of Animal Husbandry and Veterinary Medicine, Changchun, China; 3 Animal Husbandry Development Service Center of Tongyu County, Baicheng, China; 4 Animal Disease Prevention and Control Center of Tongyu County, Baicheng, China; 5 Animal Disease Prevention and Control Center, Xing’an League, China

**Keywords:** BRSV, BVDV, enzymatic recombinase amplification, ERA-LFD, lateral-flow dipsticks

## Abstract

Bovine respiratory disease (BRD) is the costliest bovine syndrome worldwide, inflicting annual losses of over one billion USD in North America alone. Transport stress, overcrowding and viral–bacterial synergy can drive mortality to 70%, yet laboratory-based diagnostics delay decisive treatment. We therefore developed pen-side real-time enzymatic recombinase amplification lateral-flow dipsticks (RT-ERA-LFD) assays targeting the two principal viral agents, bovine respiratory syncytial virus (BRSV) and bovine viral diarrhoea virus (BVDV), which enables their separate detection in a single tube. The BRSV nucleoprotein gene and BVDV 5’-UTR were cloned and in-vitro transcribed into quantified RNA standards to calibrate an enzymatic recombinase amplification (ERA) coupled with lateral-flow dipsticks (LFD). After primer/probe optimisation (BRSV-ERA-F1/R4/P2; BVDV-ERA-F1/R4/P1), the 40 °C, 20-min reactions detected as few as 10 template copies, showed 100% specificity against related bovine pathogens and matched real-time PCR results in 46 archived respiratory samples. In a field survey of nasal swabs from cattle farms in Jilin Province, China, BRSV was detected in 10.87% and BVDV in 8.70% of specimens, with results identical to qPCR obtained within 30 min without instrumentation. By delivering actionable infection status at the chute, the platform enables mass screening and timely intervention, effectively mitigating BRD’s global economic impact.

## Introduction

BRD is a complex, multifactorial syndrome that inflicts the greatest economic losses on the global cattle industry; annual costs in North America alone exceed 1 billion USD (1–3). The condition presents as acute or chronic respiratory signs triggered by environmental stress, viral–bacterial co-infections, and host susceptibility (1). Transport, overcrowding, poor ventilation, or sudden dietary changes compromise pulmonary defenses and allow pathogens to colonize the upper and lower airways. Clinically, BRD is characterized by fever, cough, nasal/ocular discharge, tachypnoea, and reduced productivity; severe cases progress to pneumonia with mortality rates up to 70% (2). Consequently, BRD is the costliest disorder encountered in feedlot and dairy operations, underscoring the need for integrated control strategies that include vaccination, biosecurity, and rapid diagnostics (3). The principal viral agents implicated in BRD are BRSV and BVDV.

BRSV belongs to the genus *Orthopneumovirus*, subfamily *Pneumovirinae*, family *Paramyxoviridae*. It is an enveloped, negative-sense, single-stranded RNA virus bearing surface glycoprotein spikes (4). Explosive outbreaks occur mainly in autumn and winter, and stress-related triggers frequently precipitate disease; secondary bacterial or viral infections can markedly increase mortality. Calves less than 6 months of age are particularly susceptible, often developing severe bronchiolitis and pneumonia (5). Typical clinical signs include depression, anorexia, pyrexia, increased respiratory rate, coughing, profuse salivation and lacrimation, reduced milk yield, and impaired semen quality in bulls. The widespread circulation of BRSV in China is thought to be linked to large-scale importation of cattle.

BVDV is a member of the genus *Pestivirus*, family *Flaviviridae*, and is an enveloped, positive-sense, single-stranded RNA virus (6). It replicates readily in fetal bovine kidney, spleen, testis, turbinate, and tracheal tissues. Based on its ability to induce cytopathic effect *in vitro*, BVDV is classified as either cytopathic (CP) or non-cytopathic (NCP) (7). The disease typically manifests acutely, with fever, serous to mucopurulent nasal discharge, anorexia, salivation, and erosions of the nasal planum and oral mucosa. A profuse, watery diarrhoea follows, often becoming mucoid and blood-tinged. Acute cases usually die within 2 weeks, whereas chronically affected animals show coalescing erosions on the nasal planum. The virus circulates year-round, peaking in late winter and early spring, and affects both pastured and housed cattle.

Both viruses are readily transmitted via aerosols and close contact and can spread rapidly within herds. Current diagnostic approaches for BRSV and BVDV rely on virus isolation, molecular assays, or serology (8); however, these methods require well-equipped laboratories, are costly and time-consuming, and are unsuitable for on-farm use. Moreover, prolonged isolation times hinder timely vaccine development. ERA, which was introduced by GenDx Biotech, was a modified version of recombinase polymerase amplification (RPA) that completes nucleic acid amplification in 30 min at 37 °C–40 °C without instrumentation (9). Results can be interpreted by real-time fluorescence, gel electrophoresis, or LFD (10), offering exceptional convenience. In recent years, isothermal amplification has been increasingly adopted for the rapid detection of animal, plant, and microbial pathogens. To ensure accuracy and reproducibility, we selected the highly conserved N gene of BRSV (11) and the 5′-UTR of BVDV (12) as targets and employed gene cloning and *in-vitro* transcription to produce quantified RNA standards. These standards will allow direct comparison with clinical specimens, ensuring reliable quantification and validation of the ERA-LFD assays. Establishing such rapid, user-friendly tests will facilitate large-scale epidemiological surveys, reduce economic losses in intensive cattle operations, and support the formulation of region-specific integrated BRD control strategies.

## Materials and methods

### Viruses and cells

BRSV and BVDV are both preserved in the Parasitology Laboratory of the Jilin Academy of Animal Science and Veterinary Medicine. DH5α competent cells were purchased from Sangon Biotech (Shanghai) Co., Ltd.

### Plasmids, reagents and genomes

The pET-28a(+) vector, QuickCut™ BamH I, QuickCut™ Hind III, and DL2000 DNA Marker were purchased from Toyobo Bio-Engineering (Dalian) Co., Ltd. The virus DNA/RNA extraction Kit was purchased from Xi’an Tianlong Technology Co., Ltd. The 2×Taq Plus PCR Master Mix, FastKing One Step RT-PCR Kit, Plasmid Mini Kit, and DNA Gel Extraction Kit were purchased from Tiangen Biotech (Beijing) Co., Ltd. The 4S Green Plus Nucleic Acid Stain, Seamless Cloning Kit, and T7 RNA In Vitro Transcription Kit were purchased from Sangon Biotech (Shanghai) Co., Ltd. The TaqMan™ Fast Virus One-Step Multiplex Master Mix was purchased from Thermo Fisher Scientific (China) Co., Ltd. The Basic Nucleic Acid Amplification Kit (ERA), RT-Basic Nucleic Acid Amplification Kit (ERA), Strip-Based Nucleic Acid Amplification Kit (ERA), RT-Strip-Based Nucleic Acid Amplification Kit (ERA), and lateral flow test strips were purchased from Suzhou GenDx Biotech Co., Ltd. Infectious bovine rhinotracheitis virus (IBRV), BRSV, BVDV, Bovine Coronavirus (BCoV), *Pasteurella multocida*, and *Toxoplasma gondii* genomes are preserved in the Parasitology Laboratory of the Jilin Academy of Animal Science and Veterinary Medicine, and 46 bovine nasal swabs were obtained from cattle farms in Changchun, Jilin Province. All clinical samples were collected from July 2023 to October 2024 and stored at 4 °C temporarily.

### Viral RNA extraction and cDNA synthesis

The viral genomes from BVDV, BRSV, and clinical samples were extracted by the Automated Nucleic Acid Extraction System. Total RNA extracted from BRSV and BVDV was reversed-transcribed into cDNA using the RevertAid First Strand cDNA Synthesis Kit (Thermo Fisher Scientific) following the manufacturer’s instructions.

### Construction of positive standard plasmids based on the BVDV N gene and the BRSV 5′-UTR

Following the kit’s instruction, reverse-transcribed BVDV cDNA and BRSV cDNA were used as templates for PCR. The 25-μL reaction contained 12.5 μL 2×Taq Plus PCR Mix, 1 μL each of primers BRSV-N-28aF (or BVDV-5UTR-28aF) and BRSV-N-28aR (or BVDV-5UTR-28aR), 3 μL of templates, and nuclease-free water to volume. Cycling conditions were: 94 °C 3 min; 30 cycles of 94 °C 30 s, 55 °C 30 s, 72 °C 1 min; final extension 72 °C 5 min; hold at 4 °C. Specific primers targeting the conserved regions of the BRSV N gene (GenBank No. NC_001989.1) and the BVDV 5′-UTR (GenBank No. KP749796.1) were designed. BamHI and HindIII restriction sites were appended to the 5′ and 3′ ends of the primers, respectively. Details are provided in Table 1.

**Table 1:** Primer sequences.

Primers	Sequences (5′ → 3′)	Tm (°C)	GC %	ΔG hairpin (kcal/mol)	ΔG dimer (kcal/mol)
BRSV-N-28aF	CAGCAAATGGGTCGCGGATCCATGGCTCTTATCAAGGTCAAA	65.2	48.6	−0.4	−1.0
BRSV-N-28aR	CTCGAGTGCGGCCGCAAGCTTTCACAATTCCACATCATTATC	64.8	45.0	−0.6	−0.8
BVDV-5UTR-28aF	CAGCAAATGGGTCGCGGATCCGCCATGCCCTTAGTAGGACTA	66.1	50.0	−0.3	−0.9
BVDV-5UTR-28aR	CTCGAGTGCGGCCGCAAGCTTCAACTCCATGTGCCATGTACA	65.5	47.4	−0.5	−0.7

Primers were designed with Primer Premier 5 (length 30–40 nt, Tm 64 °C–66 °C, GC 45%–55%, no ΔG<−1 kcal/mol secondary structures). Underlined BamHI/HindIII sites added for cloning.

The pET-28a vector was double-digested with BamHI and HindIII. PCR products and linearized vector were separated on 1% agarose gels, excised and purified. Insert fragment and vector were joined in a 20-μL reaction containing 1 μL of linearized vector, 3 μL of insert fragments, 10 μL of 2×Seamless Cloning MasterMix, and sterile ddH_2_O to volume, incubated at 50 °C for 20 min and chilled on ice for 2 min. The assembly mixture was transformed into *E. coli* DH5α competent cells; recombinants were selected by kanamycin. The pET28a-BRSV-N and pET28a-BVDV-5UTR plasmids were verified by PCR and sequencing, then quantified and stored as standard positive controls for the BVDV ERA and BRSV ERA assays.

### Homogeneity-stability verification of positive standards

After the concentration of the positive standard was determined with a UV–vis spectrophotometer, the copy number was calculated using the formula: Copy number (copies/μL) = (6.02 × 10^23^) × (ng/μL × 10^−9^) / (RNA length × 340). Homogeneity of the same batch of BRSV and BVDV positive standards was assessed by random sampling. The accurately diluted positive standard was dispensed into 100 identical aliquots; five aliquots were randomly selected and tested by qPCR. Consistent Ct values indicate good homogeneity, whereas large deviations suggest heterogeneity.

Stability under different storage temperatures was evaluated by storing the aliquoted positive standards at −80 °C and testing aliquots on days 7, 30, and 180 by qPCR. The cycle threshold (Ct) value reflecting the fluorescence signal was used to quantify the nucleic acid content and thus the stability of the standards under the specified storage conditions.

### ERA primer design and screening

Based on the conserved regions of the BRSV N gene (NC_001989.1) and the BVDV 5’-UTR sequence (KP749796.1), and according to the primer design principles of the ERA method, three forward primers, four reverse primers, and two probes were designed for BRSV, and two forward primers, four reverse primers, and three probes were designed for BVDV. The synthesis of primers and probes was completed by Sangon Biotech (Shanghai) Co., Ltd., and the sequence information is shown in Table 2. According to the instructions for the RT-ERA Basic Nucleic Acid Amplification Kit, the reaction system was set up as follows: 20 μL of solubilizing agent, 2.5 μL each of the forward and reverse primers (10 μmol/L), 2 μL of template, and ddH_2_O added to a final volume of 48 μL. After brief mixing, the mixture was added to each tube containing the basic amplification reagent. Then, 2 μL of activator was added to the inside of the tube cap. Following a quick centrifugation, the tubes were briefly vortexed and centrifuged again. The reaction conditions for BRSV and BVDV primer screening were set at 40 °C for 20 min. Next, 10 μL of the amplification product was mixed with 1.5 μL of the 6× Loading Buffer provided with the kit, incubated at 56 °C for 5 min, and then subjected to 1.5% agarose gel electrophoresis. Amplification results were observed to select optimal primer pairs. The above procedure was repeated to confirm the best primer pairs for the BRSV and BVDV RT-ERA assays.

**Table 2:** Probe sequences.

Probes	Sequence (5′ → 3′)	Tm (°C)	GC (%)	ΔG hairpin (kcal/mol)	ΔG dimer (kcal/mol)
BVDV-ERA-F1	TGAGTACAGGGTAGTCGTCAGTGGTTCG	60.1	50.0	−0.7	−1.1
BVDV-ERA-F2	TAGCAGCAGTGGCGAGTTCGTTGGGTGG	60.8	54.2	−1.3	−1.5
BVDV-ERA-R1	TTTACTCAACCACTTTCACCTGGGCGAC	59.6	46.7	−0.9	−1.2
BVDV-ERA-R2	TTTAGTAGCAACACAGTGGGCCTCTGCA	60.0	46.7	−0.8	−1.0
BVDV-ERA-R3	CCTATCAGGCCGTGTCCGTAATGGTTTA	59.2	46.7	−0.7	−1.4
BVDV-ERA-R4	TTTTAGTAGCAACACAGTGGGCCTCTGC	59.9	48.1	−0.5	−0.9
BVDV-ERA-P1	(FAM-dT)CGTGGACGAGGGCATGCCCACAGCACATCCTA(THF)CCTGGACGGGGGTCGCCC(C3-SPACER)	68.5	62.5	−0.9	−1.0
BVDV-ERA-P2	(FAM-dT)CCACGTGGACGAGGGCATGCCCACAGCACATC(THF)TAACCTGGACGGGGGT(C3-SPACER)	68.8	60.9	−1.1	−1.3
BVDV-ERA-P3	(FAM-dT)GGACTAGCATACCGGGGGGGGTAGCAACAGTG(THF)TGAGTTCGTTGGATGG(C3-SPACER)	68.2	56.5	−0.8	−1.2
BRSV-ERA-F1	TAGGTATGTTATATGCTATGTCCCGATTG	59.6	40.0	−0.6	−1.2
BRSV-ERA-F2	TATGTTATATGCTATGTCCCGATTGG	59.0	39.3	−0.7	−1.4
BRSV-ERA-F3	TTAAAATACTCAAAGATGCAGGCTACCAAG	58.9	36.7	−0.9	−1.5
BRSV-ERA-R1	CCTTGACTCTATTTCTATATTACCTTGAACTTC	59.3	36.4	−0.8	−1.3
BRSV-ERA-R2	TACCTCTCCCATCTCTTTTAGCATCTTTT	58.7	38.7	−0.7	−1.1
BRSV-ERA-R3	GACTTCCTTGACTCTATTTCTATATTACCTTGA	59.5	39.4	−0.6	−1.0
BRSV-ERA-R4	CTGATGTTAAGCTGACTAATGTTAGCACTT	58.9	42.3	−0.4	−0.8
BRSV-ERA-P1	(FAM-dT)TGCAGGCTACCAAGTGAGGGCCAATGGGGTTG(THF)TGTGATAACACATCGA(C3-SPACER)	68.4	50.0	−1.0	−1.1
BRSV-ERA-P2	(FAM-dT)TGGGGTTGATGTGATAACACATCGACAGGAT(THF)TGAATGGAAAAGAAAT(C3-SPACER)	69.0	41.7	−0.8	−1.1

THF, tetrahydrofuran spacer; C3, C3-spacer blocking elongation.

### Development of the ERA-LFD detection method and probe screening

The selected optimal forward and reverse primers were synthesized by Sangon Biotech, with a Biotin group added to the 5′ end of the reverse primer. Probe screening was then performed separately for BRSV and BVDV using the RT-ERA Dipstick Nucleic Acid Amplification Kit, following the manufacturer’s instructions. The previously prepared positive standards for BRSV and BVDV were used as templates, and a negative control was included in each assay. The reaction mixture consisted of 20 μL solubilizing agent, 2.1 μL each of forward and reverse primers (10 μmol/L), 0.6 μL of probe, 2 μL of template, and ddH_2_O added to a final volume of 48 μL. After brief mixing, the mixture was added to the RT-dipstick amplification reagent. Then, 2 μL of activator was carefully pipetted into the tube cap. The tubes were subjected to a quick spin, briefly vortexed, and centrifuged again. The reaction conditions for probe screening were 40 °C for 30 min. After amplification, each product was diluted 80-fold in a buffer containing 0.1% Tween-20. An 100 μL aliquot of the diluted product was applied to the sample pad of a lateral-flow dipstick (Suzhou GenDx Biotech) and incubated at room temperature (22 °C–25 °C). Results were read at 5 min; strips interpreted after 15 min were considered invalid. A valid test was scored as follows: positive: both control (C) and test (T) lines clearly visible; negative: only C line visible; invalid: C line absent, requiring retesting.

### Optimization of the ERA-LFD detection platform

For both the BRSV RT-ERA-LFD and BVDV RT-ERA-LFD assays, the reaction temperature was systematically varied—39 °C, 40 °C, 41 °C, 42 °C and 43 °C—while the incubation time was kept at 30 min. After amplification, each product was diluted 80-fold; 100 μL of the diluted solution was transferred to a 1.5 mL microtube, a lateral-flow dipstick was inserted, and the result was read within 10 min. By comparing the LFD signals across the temperature gradient, the optimal reaction temperature for each ERA-LFD method was selected.

For both the BRSV and BVDV RT-ERA-LFD assays, the incubation time was tested at 10, 15, 20, 25, 30, 35 and 40 min while the reaction temperature was fixed at the optimum value determined previously. After amplification, each product was diluted 80-fold; 100 μL of the diluted solution was transferred to a 1.5 mL microtube, a lateral-flow dipstick was inserted, and the result was read within 10 min. By comparing the LFD signals across the time-course, the optimal reaction time for each ERA-LFD method was selected.

### Analytical performance evaluation

The specificity of the established ERA-LFD assays was evaluated using nucleic acids from IBRV, BRSV, BVDV, BCoV, *Pasteurella multocida* (*P. multocida*) and *Toxoplasma gondii* (*T. gondii*), with ddH_2_O as the blank control, to confirm that both the BRSV and BVDV RT-ERA-LFD methods recognize only their intended targets.

The BRSV and BVDV positive standards were first adjusted to 10^8^ copies/μL and then serially diluted 10-fold. Each dilution was tested with the newly established BRSV RT-ERA-LFD and BVDV RT-ERA-LFD assays. After amplification, the lateral-flow dipsticks were read and the lowest dilution that still gave a visible positive signal was taken as the limit of detection (LOD) for each method. Using BVDV and BRSV positive standards as templates, amplification was carried out with the PCR method already established in the laboratory. The products were analyzed by 1% agarose gel electrophoresis to compare the sensitivity of the methods. The specific primers are listed in Table 3. The RT-PCR for BRSV and BVDV was carried out in a 50 μL mixture containing 25 μL of 2× FastKing One-Step RT-PCR MasterMix, 2 μL of 25× RT-PCR Enzyme Mix, 1.25 μL each of forward and reverse primers (10 μM), 2 μL of template RNA, and RNase-free ddH_2_O to volume. Cycling conditions were: 42 °C for 30 min (reverse transcription); 95 °C for 3 min (initial denaturation); 35 cycles of 94 °C for 30 s, 55 °C for 30 s, and 72 °C for 30 s; final extension at 72 °C for 5 min; hold at 4 °C.

**Table 3:** Primers for sensitivity evaluation.

Primers	Sequence (5′ → 3′)	Size (bp)	Tm (°C)	GC (%)	ΔG hairpin (kcal/mol)	ΔG dimer (kcal/mol)
BRSV-F	TCACTGCAGTCATTAGGAGAGC	526	56.4	47.6	−0.5	−1.0
BRSV-R	GCATATGCTTTGGCAGCATC		56.0	45.0	−0.7	−0.9
BVDV-F	CTAGCAAAATGAGGGGGGTAG	266	57.1	47.6	−0.6	−1.1
BVDV-R	CATGTGCCATGTACAGCAGAG		57.3	52.4	−0.4	−0.8

Primers designed for routine RT-PCR (length 20–22 nt, Tm 56 °C–58 °C, GC 45%–55%, no predicted strong secondary structures).

A total of 26 nasal-swab specimens collected from cattle herds in Changchun, suspected of BRSV/BVDV infection, were tested in parallel with the newly developed ERA-LFD assay and the conventional RT-PCR protocol already established in our laboratory. Concordance between the two methods was calculated from Table 4 as: Concordance (%) = (a + d)/(a + b + c + d) × 100. The two-by-two table was defined with RT-PCR as the gold standard: a) both ERA-LFD and RT-PCR positive; b) ERA-LFD positive but RT-PCR negative; c) ERA-LFD negative but RT-PCR positive; d) both negative. RT-PCR positivity required Ct ≤ 35 with a single melting-curve peak, while ERA-LFD positivity was defined by the visibility of both the test (T) and control (C) lines. Concordance was assessed using the overall percent agreement [OPA = (a + d)/*N* × 100%] and Cohen’s *κ* coefficient [κ = (Po–Pe)/(1–Pe)], with κ > 0.80 indicating excellent agreement. All amplicons that gave a positive signal in the ERA-LFD assay were sent to Sangon Biotech for sequencing to confirm their identity and accuracy.

**Table 4:** Compliance rate.

		RT-PCR		Total
		+	−	
ERA-LFD	+	a	b	a + b
	−	c	d	c + d
Total		a + c	b + d	a + b + c + d

The two-by-two table was defined with RT-PCR as the gold standard: a) both ERA-LFD and RT-PCR positive; b) ERA-LFD positive but RT-PCR negative; c) ERA-LFD negative but RT-PCR positive; d) both negative. RT-PCR positivity required Ct ≤ 35 with a single melting-curve peak, while ERA-LFD positivity was defined by the visibility of both the test (T) and control (C) lines. Concordance was assessed using the overall percent agreement [OPA = (a + d)/*N* × 100%] and Cohen’s κ coefficient [κ = (Po–Pe)/(1–Pe)], with κ > 0.80 indicating excellent agreement.

### Clinical sample detection

A total of 920 cattle from farms and slaughterhouses in five areas of Jilin Province—Nong’an, Baicheng, Dunhua, Da’an, and Changchun—were sampled at 5%, yielding 46 nasal swabs. We determined a sampling rate of 5% (46 out of 920 head) by considering the available on-site manpower, which included two veterinarians capable of collecting up to 20 samples per day. This rate also met the minimum requirement of 45 samples to achieve an expected prevalence of 8%–11% with 95% confidence, as calculated using Epitools. Additionally, this approach was chosen to minimize stress associated with transportation. Nucleic acids were extracted and tested with the field-assembled BRSV RT-ERA-LFD and BVDV RT-ERA-LFD kits, and every result was cross-verified with the laboratory-established qPCR assay (see Table 5).

**Table 5:** Demographic characteristics of the 46 cattle enrolled in the field evaluation of RT-ERA-LFD.

Item	Category	Number	Proportion (%)
Age	<6 months	18	39.1
	6–12 months	20	43.5
	>12 months	8	17.5
Breed	Simmental cross	28	60.9
	Holstein	12	26.1
	Yanbian Yellow	6	13.0
Health*	Clinically healthy	40	87.0
	Mild nasal discharge	5	10.9
	Moderate respiratory signs	1	2.1

*No animal had rectal temperature >40 °C.

## Results

### Identification of standard positive plasmids

**Figure 1:**
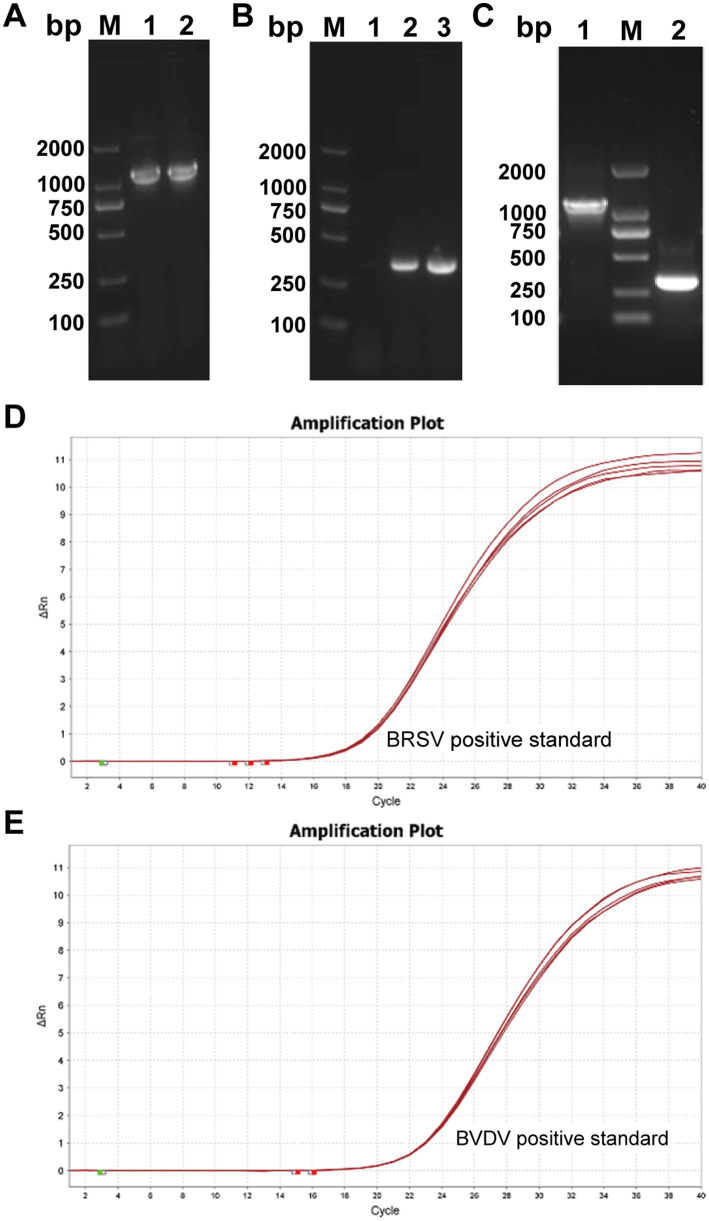
Construction of standard positive plasmids. **(A)** Visualization of BRSV-N gene amplification by gel electrophoresis. **(B)** Visualization of BVDV-5UTR amplification by gel electrophoresis. **(C)** Verification of the recombinant plasmid pET-28a-BRSV-N/BVDV-5UTR. Using the extracted plasmids as templates, PCR identification yielded target fragments of the expected sizes (1,176 bp for BRSV-N and 290 bp for BVDV-5UTR). **(D,E)** Homogeneity of positive BRSV **(D)** and BVDV **(E)** positive standards was assessed by qPCR.

Using the cDNA reverse-transcribed from BRSV and BVDV RNA as templates, two pairs of gene-specific primers (BRSV-N-28aF/R and BVDV-5UTR-28aF/R) were employed in independent PCRs. Amplicons of 1,176 bp and 290 bp, corresponding to the predicted sizes, were obtained (Figures 1A,B). The purified BRSV-N and BVDV-5UTR fragments were ligated into pET-28a previously digested with the appropriate restriction enzymes. After transformation into *E. coli*, single colonies were picked and plasmid DNA was extracted. PCR screening of the resulting clones yielded products identical in size to the original inserts (Figure 1C). Sanger sequencing confirmed 100% nucleotide identity with the target sequences.

### Homogeneity–stability of positive standards

*In-vitro*-transcribed RNAs corresponding to the BVDV-5UTR and BRSV-N genes were quantified spectrophotometrically. The BRSV standard contained 36.864 ng/μL, whereas the BVDV standard contained 15.130 ng/μL. Using the molecular weight of single-stranded RNA, these concentrations correspond to 5.55 × 10^10^ copies/μL and 9.23 × 10^10^ copies/μL, respectively. Each standard was divided into 100 aliquots of equal volume; five aliquots were then selected at random and tested by one-step qPCR to assess homogeneity. Ct values ranged from 11.17 to 13.05 for the BRSV standard (Figure 1D) and from 15.12 to 16.03 for the BVDV standard (Figure 1E). The coefficient of variation (CV) was 6.35% for BRSV and 2.53% for BVDV, indicating low inter-aliquot variability and high uniformity. Thus, both RNA standards are sufficiently homogeneous for use as quantitative reference materials in downstream assays.

To assess the long-term stability of the positive standards, aliquots stored at −80 °C were analyzed by qPCR on days 7, 30, and 180 post-freezing. No significant differences were observed in the amplification curves across the tested time points. The CV for Ct values were 4.17% for the BRSV standard and 3.31% for the BVDV standard (Supplementary Table 1). These results demonstrate that both BRSV and BVDV positive standards maintain good stability when stored at −80 °C for up to 6 months.

### The optimal primer pairs and probes for BRSV and BVDV RT-ERA-LFD

**Figure 2:**
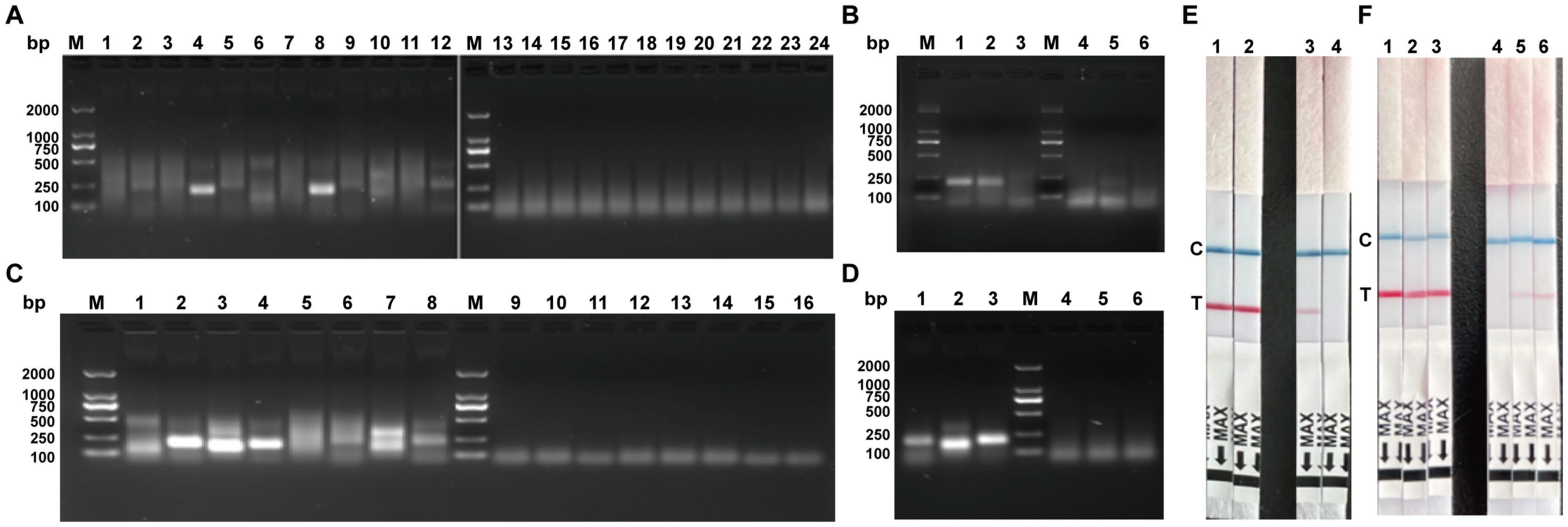
Screening of the optimal primer pair for RT-ERA and determination of the optimal RT-ERA–LFD probe. **(A)** Screening of BRSV RT-ERA primer pairs. M: DNA marker DL2000; 1–12: The amplification results of 12 primer pairs BRSV-ERA-F1/R1 to BRSV-ERA-F3/R4; 13–24: Negative controls for amplification with 12 primer pairs BRSV-ERA-F1/R1–BRSV-ERA-F3/R4. **(B)** Screening of BRSV RT-ERA optimal primer pair. M: DNA marker DL2000; 1–3: Amplification results of primer pairs BRSV-ERA-F1/R4, BRSV-ERA-F2/R4, and BRSV-ERA-F3/R4; 4–6: Negative controls for amplification with primer pairs BRSV-ERA-F1/R4, BRSV-ERA-F2/R4, and BRSV-ERA-F3/R4. **(C)** Screening of BVDV RT-ERA primer pairs. M: DNA marker DL2000; 1–8: Amplification results of 8 primer pairs BVDV-ERA-F1/R1–BVDV-ERA-F2/R4; 9–16: Negative controls for amplification with 8 primer pairs BVDV-ERA-F1/R1–BVDV-ERA-F2/R4. **(D)** Screening of BVDV RT-ERA optimal primer pair. M: DNA marker DL2000; 1–3: Amplification results of primer pairs BVDV-ERA-F1/R2, BVDV-ERA-F1/R3, and BVDV-ERA-F1/R4; 4–6: Negative controls for amplification with primer pairs BVDV-ERA-F1/R2, BVDV-ERA-F1/R3, and BVDV-ERA-F1/R4. **(E)** Screening of the optimal probe for BRSV RT-ERA-LFD. 1: BRSV-ERA-P1; 2: BRSV-ERA-P2; 3: Negative control for BRSV-ERA-P1; 4: Negative control for BRSV-ERA-P2. **(F)** Screening of the optimal probe for BVDV RT-ERA-LFD. 1: BVDV-ERA-P1; 2: BVDV-ERA-P2; 3: BVDV-ERA-P3; 4: Negative control for BVDV-ERA-P1; 5: Negative control for BVDV-ERA-P2; 6: Negative control for BVDV-ERA-P3.

Using the previously prepared BRSV-positive standard as template, 12 primer pairs (BRSV-ERA-F1/R1–F3/R4) were screened by RT-ERA; the two that yielded visible amplicons—BRSV-ERA-F1/R4 and F2/R4 —were retested (Figures 2A,B), and BRSV-ERA-F1/R4 produced the brightest, clearest band, designating F1/R4 as the optimal BRSV pair. Similarly, with the BVDV-positive standard, eight primer pairs (BVDV-ERA-F1/R1–F2/R4) were evaluated; BVDV-ERA-F1/R2, F1/R3 and F1/R4 gave visible products (Figure 2C), and subsequent retesting (Figure 2D) showed BVDV-ERA-F1/R4 to be the brightest and most specific, establishing BVDV-ERA-F1/R4 as the optimal BVDV primer pair. Using BRSV-positive RNA and primers F1/R4, dipstick screening (Figure 2E) showed P1 and P2 both positive; however, P1 produced a false-positive in the negative control, so P2 was selected. For BVDV (Figure 2F), P1–P3 all generated positive signals, yet only P1’s negative control remained clean, so P1 was chosen as the optimal BVDV probe.

### Optimal reaction condition

**Figure 3:**
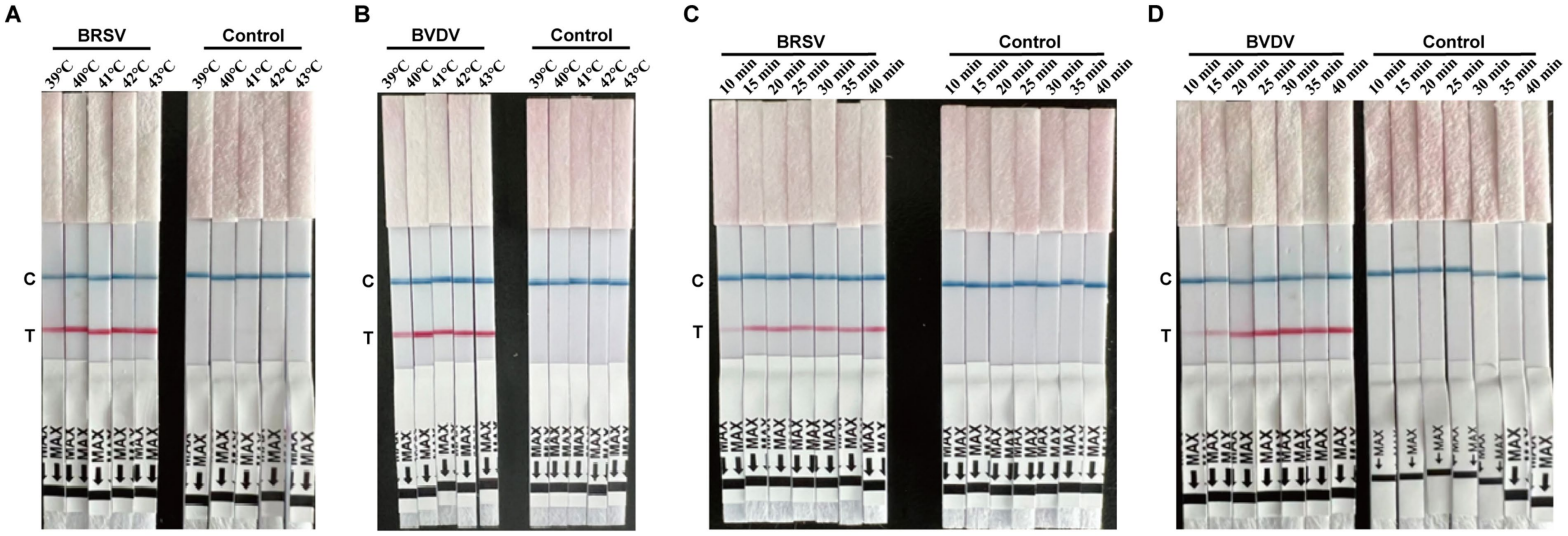
Optimization of the BRSV and BVDV RT-ERA-LFD platform conditions. **(A,B)** Optimization of the RT-ERA-LFD reaction temperature for BRSV and BVDV. Temperature gradients of 39 °C, 40 °C, 41 °C, 42 °C, and 43 °C were tested for BRSV **(A)** and BVDV **(B)**, with corresponding negative controls. **(C,D)** Optimization of the RT-ERA-LFD reaction time for BRSV and BVDV. Time gradients of 10, 15, 20, 25, 30, 35 and 40 min were tested for BRSV **(C)** and BVDV **(D)**, with corresponding negative controls.

For both the BRSV and BVDV RT-ERA-LFD assays, reactions were run at 39 °C, 40 °C, 41 °C, 42 °C, and 43 °C. As shown in Figure 3A (BRSV) and Figure 3B (BVDV), distinct control (C) and test (T) lines were observed at every temperature. Because the kit specifications recommend 40 °C–42 °C and a lower temperature reduces energy consumption, 40 °C was selected as the optimal reaction temperature. Using this temperature, amplification times of 10, 15, 20, 25, 30, 35 and 40 min were then compared (Figures 3C,D). All durations produced clear C and T lines; however, to meet the requirements of rapid clinical testing, 20 min was chosen as the standard reaction time for both assays.

### Analytical performance results

**Figure 4:**
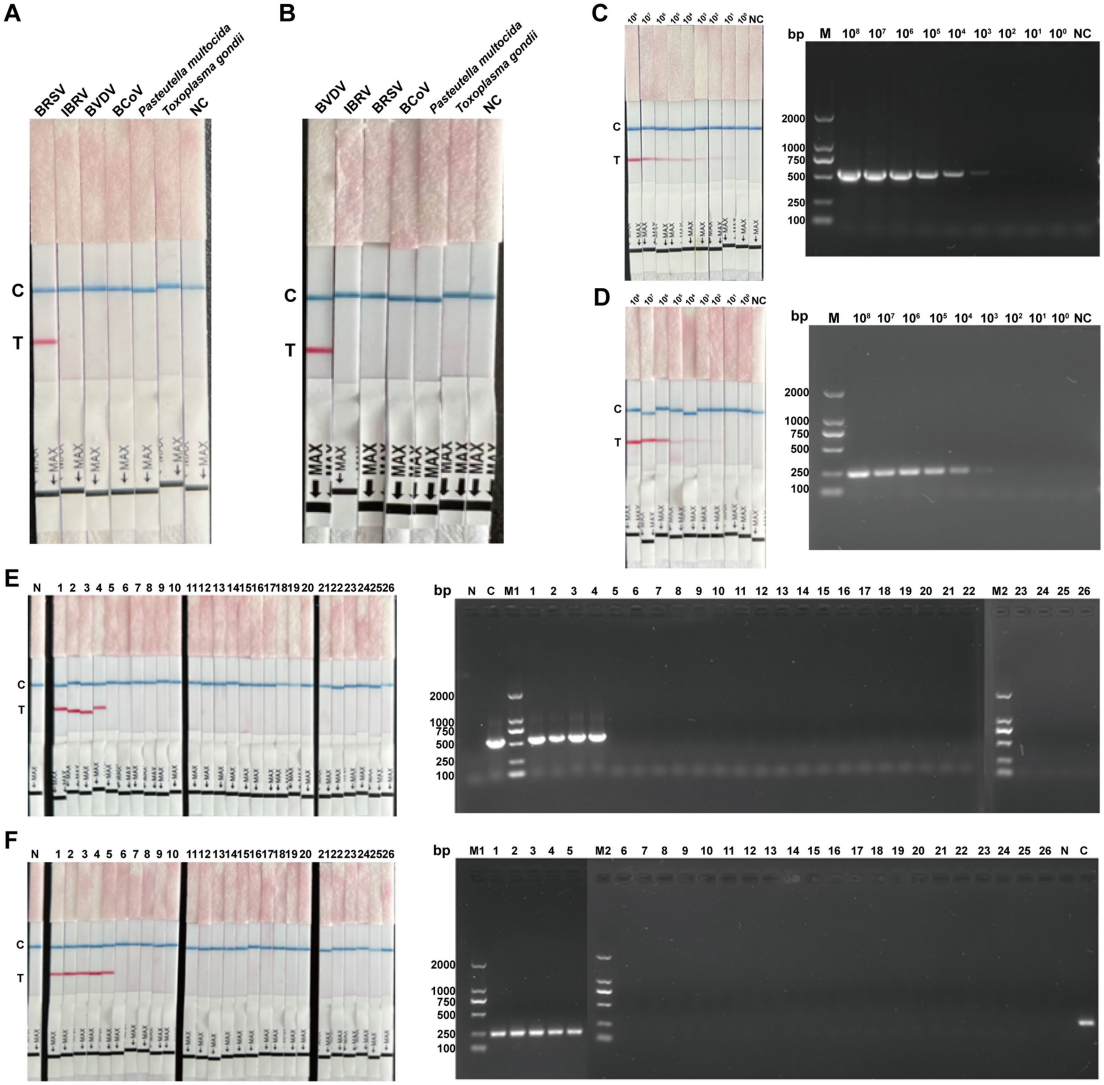
Evaluation of sensitivity, specificity, and concordance of the detection platform. **(A,B)** Specificity evaluation of BRSV and BVDV RT-ERA-LFD assays. Genomic materials of BRSV, BVDV, IBRV, BCoV, *P. multocida* and *T. gondii* archived in our laboratory were used for specificity testing. In **(A)**, lanes 1–7 contain BRSV, IBRV, BVDV, BCoV, *P. multocida*, *T. gondii* and a negative control; in **(B)** the order is BVDV, IBRV, BRSV, BCoV, *P. multocida*, *T. gondii* and negative control. **(C)** Sensitivity evaluation of BRSV RT-ERA-LFD (left) and RT-PCR (right). In the left panel, lanes contain 10^8^–10^0^ copies/μL BRSV RNA standard followed by the negative control; in the right panel, the same samples are preceded by the DNA DL2000 marker. **(D)** Sensitivity evaluation of BVDV RT-ERA-LFD (left) and RT-PCR (right). In the left panel, lanes contain 10^8^–10^0^ copies/μL BVDV RNA standard followed by the negative control; in the right panel, the same samples are preceded by the DNA DL2000 marker. **(E)** Concordance testing of BRSV RT-ERA-LFD (left) versus RT-PCR (right). Left panel: 26 suspected samples plus negative control. Right panel: DNA DL2000 marker, the same 26 suspected samples, and both positive and negative controls. **(F)** Concordance testing of BVDV RT-ERA-LFD (left) versus RT-PCR (right). Left panel: 26 suspected samples plus negative control. Right panel: DNA DL2000 marker, the same 26 suspected samples, and both positive and negative controls.

To assess specificity, the established BRSV and BVDV RT-ERA-LFD assays were challenged with IBRV, BCoV, *P. multocida*, *T. gondii* and the homologous viruses. Only the respective target virus (BRSV or BVDV) generated a red test line (T); all other samples showed only the control line (C) (Figures 4A,B), confirming high specificity for both methods.

To evaluate sensitivity, serial 10-fold dilutions of BRSV and BVDV RNA (10^8^–10^0^ copies/μL) were tested by the established RT-ERA-LFD assays and conventional RT-PCR. As shown in Figures 4C,D, the lower limits of detection were 10^1^ copies/μL for BRSV RT-ERA-LFD and 10^2^ copies/μL for BVDV RT-ERA-LFD, whereas RT-PCR detected 10^3^ copies/μL for both viruses. Thus, the BRSV and BVDV RT-ERA-LFD methods exhibited 100-fold and 10-fold higher sensitivity than RT-PCR, respectively.

To determine the concordance of the assays, 26 nasal swabs from suspected cases were simultaneously tested by BRSV RT-ERA-LFD, BVDV RT-ERA-LFD, and conventional RT-PCR. BRSV RT-ERA-LFD identified 4 positives (15.38%, 4/26), in full agreement with the 4 detected by RT-PCR (Figure 4E), yielding 100% concordance. Similarly, BVDV RT-ERA-LFD detected 5 positives (19.23%, 5/26), matching exactly the 5 found by RT-PCR (Figure 4F), again with 100% concordance. For BRSV, 5 samples were true-positive and 41 were true-negative, with no false-positive or false-negative results, yielding an OPA of 100% and a Cohen’s *κ* of 1.00 (95% CI: 1.00–1.00). Likewise, for BVDV, 4 true-positive and 42 true-negative samples were identified, giving identical agreement statistics (OPA = 100%, κ = 1.00; 95% CI: 1.00–1.00), indicating perfect concordance between the ERA-LFD assay and qPCR. All RT-ERA-LFD amplicons from positive samples were sequenced and confirmed as BRSV or BVDV, respectively.

### Clinical sample detection

**Figure 5:**
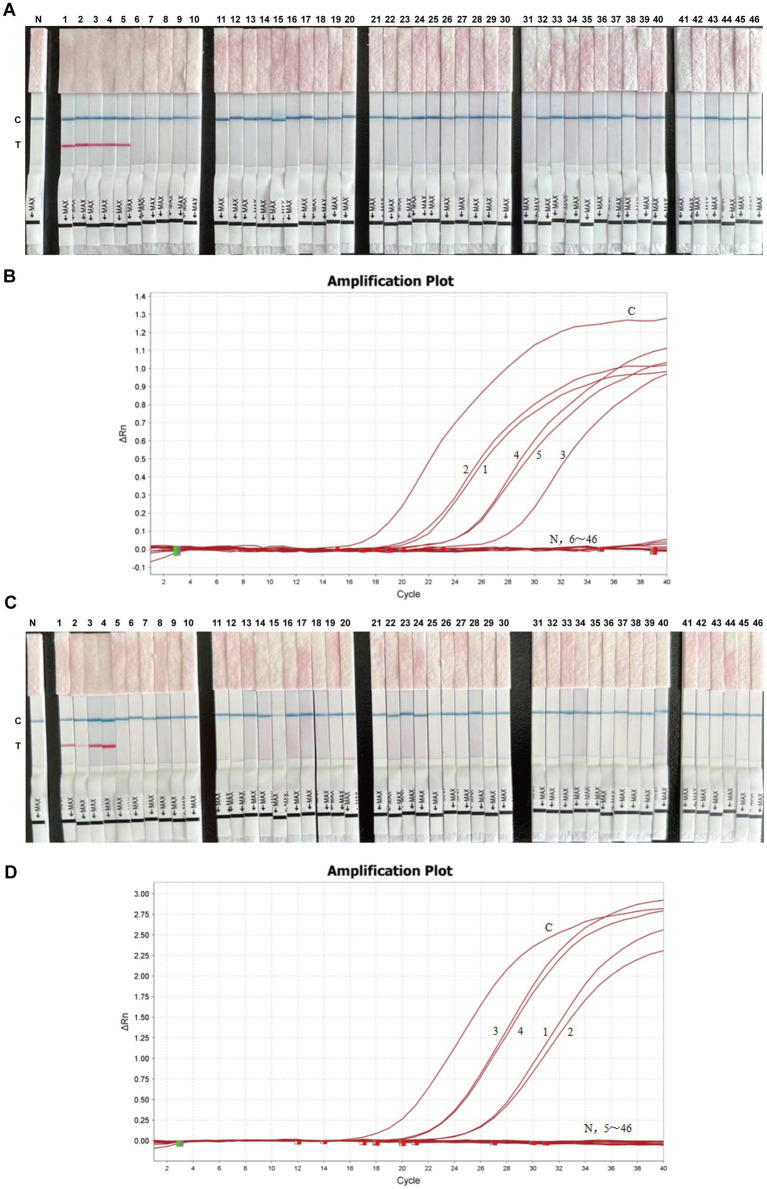
Detection of clinical samples using BRSV RT-ERA-LFD and BVDV RT-ERA-LFD. **(A)** BRSV RT-ERA-LFD results for 46 clinical samples. **(B)** qPCR detection of BRSV in the same 46 samples. **(C)** BVDV RT-ERA-LFD results for the 46 clinical samples. **(D)** qPCR detection of BVDV in the 46 samples. In each panel, C indicates the positive control and N the negative control.

The established BRSV and BVDV RT-ERA-LFD assays were used to test 46 nasal swabs collected from five prefectures in Jilin Province (Nong’an, Baicheng, Dunhua, Da’an and Changchun). Results were compared with laboratory-developed BRSV and BVDV real-time RT-PCR. BRSV RT-ERA-LFD identified five positive samples (10.87%; Figure 5A), identical to the real-time RT-PCR result (Figure 5B). BVDV RT-ERA-LFD detected four positives (8.7%; Figure 5C), again in complete agreement with the real-time RT-PCR result (Figure 5D). These data demonstrate that the newly developed RT-ERA-LFD protocols are reliable for routine clinical detection of both viruses.

## Discussion

BRSV and BVDV are the two most common infectious pathogens in cattle. BRSV typically occurs in explosive outbreaks, mainly in autumn and winter, showing a distinct seasonal pattern. Stress factors can trigger the disease, and secondary bacterial or viral infections often result in high mortality. BVDV can establish persistent infection and suppress the immune system after entering the host, creating favorable conditions for other viruses to invade. Therefore, early and rapid diagnosis of mixed infections with BRSV and BVDV is essential; although this study establishes separate single-plex ERA-LFD assays for each virus, future work will focus on integrating them into a truly multiplexed detection system.

A variety of methods—including virus isolation, multiplex PCR, real-time PCR, nested PCR and immunohistochemistry (IHC)—have been developed for BRSV and BVDV detection, yet RT-qPCR remains the gold standard because of its superior sensitivity and quantitative capacity (13). In recent years, several isothermal amplification platforms have matched qPCR in sensitivity while offering instrument-free, visual, and pen-side advantages for rapid detection of livestock pathogens. For bovine respiratory viruses, real-time reverse-transcription recombinase-aided amplification (RT-RAA) can identify BRSV within 30 min with qPCR-comparable sensitivity (14); coupling RT-RAA to a lateral-flow biosensor (LFB) permits simultaneous visual screening of BRSV and BPIV3 without a thermal cycler, greatly increasing on-farm testing throughput (15). Likewise, recombinase polymerase amplification (RPA) and its derivatives have been validated for a variety of animal pathogens. At 38 °C for 30 min, RPA-LFD and exo-RPA detect *E. miricola* down to 10^2^ copies/μL—tenfold more sensitive than conventional PCR (16). In aquatic animal virology, RPA, real-time RPA and RPA-LFD assays for LYCIV all display high specificity; the RPA-LFD format achieves a limit of detection (LOD) of 10^1^ copies/μL and does not cross-react with common aquatic pathogens (17). An RT-RPA-LFD targeting the N gene of porcine epidemic diarrhoea virus (PEDV) also exhibits specific detection at 10^2^ copies/μL without cross-amplification from major swine pathogens (18).

Building on this technical foundation, Wang et al. established an RPA-LFD assay for bovine viral diarrhoea virus (BVDV) that operates at a mild 35 °C and completes amplification in 25 min, with a limit of detection (LOD) of 60 copies μL^−1^, high specificity, and >95% concordance with RT-qPCR; the entire pen-side workflow is finished within 30 min (19, 20). Advancing beyond RPA, we developed an ERA-LFD platform capable of separately detecting BRSV and BVDV in a single tube. The ERA-LFD platform developed in this study enables the detection of BRSV or BVDV within 20 min at 40 °C, reducing the reaction time by an additional 20% and lowering the detection limit to 10–100 copies, which is 2- to 6-fold more sensitive than the previous assay. The reagents are formulated as domestically produced lyophilized spheres that maintain full activity during storage and transport at ambient temperature, thereby eliminating the necessity for precise temperature control in field conditions. The entire “sample-to-answer” workflow is completed within 30 min, providing a faster, more sensitive, robust, and cost-effective solution for large-scale, on-farm screening. By delivering an immediate infection status at the chute, the system enables mass screening and prompt intervention, mitigating the global economic impact of bovine respiratory disease.

Nevertheless, several aspects of our platform still warrant refinement. The current reaction temperature of 40 °C must be controlled with precision, complicating on-site deployment; future work should therefore shift the optimum to 37 °C—or ideally to ambient temperature—to eliminate the need for portable heaters. In addition, the present assay interrogates only a single pathogen (BRSV or BVDV). As Wang et al. demonstrated for BVDV and IBRV, a multiplex format that simultaneously detects two or more viruses would markedly increase operational efficiency. This reflects a broader challenge in point-of-care diagnostics: to keep pace with the complex etiology of field outbreaks, tests must eventually encompass bacteria, viruses and even parasites within a single reaction. Achieving such universality, however, greatly complicates target selection, primer-probe balance and dose-ranging, and will be a major focus of subsequent optimisation. Equally, although RPA-CRISPR systems are maturing rapidly, they remain largely pre-commercial because the total workflow still exceeds 1 hour and the cost per test is no less than US $5—limitations that are unacceptable for large-scale herd screening.

Given the foundational nature of the current work, multi-laboratory, high-volume studies are now imperative to rigorously evaluate robustness and reproducibility. Beyond mere parameter tuning, future trials must be conducted across diverse production systems, climatic zones and circulating strains, expanding both sample size and genetic diversity to generate the comprehensive data sets required for standardization and widespread implementation.

## Data Availability

All original findings of this study are fully contained within the article and its Supplementary files; further requests should be addressed to the corresponding authors.
