# Supplementary material for: Establishment and field validation of a rapid on-site recombinase polymerase amplification–lateral flow assay for BRSV and BVDV
Source: Front Vet Sci. 2026 Feb 27;13:1754704. doi: 10.3389/fvets.2026.1754704 (PMC12982014; doi:10.3389/fvets.2026.1754704)
Supplement: Supplementary file 1 [file Table_1.docx]

**Table 1 Primer sequences**

| **Primers** | **Sequences** **(5'→3')** | **Tm (℃)** | **GC %** | **ΔG hairpin (kcal/mol)** | **ΔG dimer (kcal/mol)** |
| --- | --- | --- | --- | --- | --- |
| BRSV-N-28aF | CAGCAAATGGGTCGCGGATCCatggctcttatcaaggtcaaa | 65.2 | 48.6 | –0.4 | –1.0 |
| BRSV-N-28aR | CTCGAGTGCGGCCGCAAGCTTTCACAATTCCACATCATTATC | 64.8 | 45.0 | –0.6 | –0.8 |
| BVDV-5UTR-28aF | CAGCAAATGGGTCGCGGATCCgccatgcccttagtaggacta | 66.1 | 50.0 | –0.3 | –0.9 |
| BVDV-5UTR-28aR | CTCGAGTGCGGCCGCAAGCTTCAACTCCATGTGCCATGTACA | 65.5 | 47.4 | –0.5 | –0.7 |

Note: Primers were designed with Primer Premier 5 (length 30-40 nt, Tm 64-66 ℃, GC 45-55 %, no ΔG<-1 kcal/mol secondary structures). Underlined BamHI/HindIII sites added for cloning.

**Table 2 Probe sequences**

| **Probes** | **Sequence (5'→3')** | **Tm (℃)** | **GC (%)** | **ΔG hairpin (kcal/mol)** | **ΔG dimer (kcal/mol)** |
| --- | --- | --- | --- | --- | --- |
| BVDV-ERA-F1 | TGAGTACAGGGTAGTCGTCAGTGGTTCG | 60.1 | 50.0 | –0.7 | –1.1 |
| BVDV-ERA-F2 | TAGCAGCAGTGGCGAGTTCGTTGGGTGG | 60.8 | 54.2 | –1.3 | –1.5 |
| BVDV-ERA-R1 | TTTACTCAACCACTTTCACCTGGGCGAC | 59.6 | 46.7 | –0.9 | –1.2 |
| BVDV-ERA-R2 | TTTAGTAGCAACACAGTGGGCCTCTGCA | 60.0 | 46.7 | –0.8 | –1.0 |
| BVDV-ERA-R3 | CCTATCAGGCCGTGTCCGTAATGGTTTA | 59.2 | 46.7 | –0.7 | –1.4 |
| BVDV-ERA-R4 | TTTTAGTAGCAACACAGTGGGCCTCTGC | 59.9 | 48.1 | –0.5 | –0.9 |
| BVDV-ERA-P1 | （FAM-dT）CGTGGACGAGGGCATGCCCACAGCACATCCTA（THF）CCTGGACGGGGGTCGCCC（C3-SPACER） | 68.5 | 62.5 | –0.9 | –1.0 |
| BVDV-ERA-P2 | （FAM-dT）CCACGTGGACGAGGGCATGCCCACAGCACATC（THF）TAACCTGGACGGGGGT（C3-SPACER） | 68.8 | 60.9 | –1.1 | –1.3 |
| BVDV-ERA-P3 | （FAM-dT）GGACTAGCATACCGGGGGGGGTAGCAACAGTG（THF）TGAGTTCGTTGGATGG（C3-SPACER） | 68.2 | 56.5 | –0.8 | –1.2 |
| BRSV-ERA-F1 | TAGGTATGTTATATGCTATGTCCCGATTG | 59.6 | 40.0 | –0.6 | –1.2 |
| BRSV-ERA-F2 | TATGTTATATGCTATGTCCCGATTGG | 59.0 | 39.3 | –0.7 | –1.4 |
| BRSV-ERA-F3 | TTAAAATACTCAAAGATGCAGGCTACCAAG | 58.9 | 36.7 | –0.9 | –1.5 |
| BRSV-ERA-R1 | CCTTGACTCTATTTCTATATTACCTTGAACTTC | 59.3 | 36.4 | –0.8 | –1.3 |
| BRSV-ERA-R2 | TACCTCTCCCATCTCTTTTAGCATCTTTT | 58.7 | 38.7 | –0.7 | –1.1 |
| BRSV-ERA-R3 | GACTTCCTTGACTCTATTTCTATATTACCTTGA | 59.5 | 39.4 | –0.6 | –1.0 |
| BRSV-ERA-R4 | CTGATGTTAAGCTGACTAATGTTAGCACTT | 58.9 | 42.3 | –0.4 | –0.8 |
| BRSV-ERA-P1 | （FAM-dT）TGCAGGCTACCAAGTGAGGGCCAATGGGGTTG（THF）TGTGATAACACATCGA（C3-SPACER） | 68.4 | 50.0 | –1.0 | –1.1 |
| BRSV-ERA-P2 | （FAM-dT）TGGGGTTGATGTGATAACACATCGACAGGAT（THF）TGAATGGAAAAGAAAT（C3-SPACER） | 69.0 | 41.7 | –0.8 | –1.1 |

Note: THF = tetrahydrofuran spacer; C3 = C3-spacer blocking elongation.

**Table 3 Primers for sensitivity evaluation**

| **Primers** | **Sequence (5'→3')** | **Size (bp)** | **Tm (****℃)** | **GC (%)** | **ΔG hairpin (kcal/mol)** | **ΔG dimer (kcal/mol)** |
| --- | --- | --- | --- | --- | --- | --- |
| BRSV-F | tcactgcagtcattaggagagc | 526 | 56.4 | 47.6 | –0.5 | –1.0 |
| BRSV-R | gcatatgctttggcagcatc |  | 56.0 | 45.0 | –0.7 | –0.9 |
| BVDV-F | ctagcaaaatgaggggggtag | 266 | 57.1 | 47.6 | –0.6 | –1.1 |
| BVDV-R | catgtgccatgtacagcagag |  | 57.3 | 52.4 | –0.4 | –0.8 |

Note：Primers designed for routine RT-PCR (length 20-22 nt, Tm 56-58 ℃, GC 45-55 %, no predicted strong secondary structures).

**Table 4 Compliance rate**

|  | | | **RT-PCR** | | **Total** |
| --- | --- | --- | --- | --- | --- |
|  |  |  | **+** | **-** |  |
| **ERA-LFD** | | + | a | b | a+b |
|  |  | - | c | d | c+d |
| **Total** |  | | a+c | b+d | a+b+c+d |

**Note:** The two-by-two table was defined with RT-PCR as the gold standard: a) both ERA-LFD and RT-PCR positive; b) ERA-LFD positive but RT-PCR negative; c) ERA-LFD negative but RT-PCR positive; d) both negative. RT-PCR positivity required Ct ≤ 35 with a single melting-curve peak, while ERA-LFD positivity was defined by the visibility of both the test (T) and control (C) lines. Concordance was assessed using the overall percent agreement (OPA = (a+d)/N × 100 %) and Cohen’s κ coefficient (κ = (Po–Pe)/(1–Pe)), with κ > 0.80 indicating excellent agreement.

**Table 5. Demographic characteristics of the 46 cattle enrolled in the field evaluation of RT-ERA-LFD**

| Item | Category | Number | Proportion (%) |
| --- | --- | --- | --- |
| Age | < 6 months | 18 | 39.1 |
|  | 6-12 months | 20 | 43.5 |
|  | > 12 months | 8 | 17.5 |
| Breed | Simmental cross | 28 | 60.9 |
|  | Holstein | 12 | 26.1 |
|  | Yanbian Yellow | 6 | 13.0 |
| Health* | Clinically healthy | 40 | 87.0 |
|  | Mild nasal discharge | 5 | 10.9 |
|  | Moderate respiratory signs | 1 | 2.1 |

*No animal had rectal temperature > 40 °C.
